# Online Control of *Lemna minor* L. Phytoremediation: Using pH to Minimize the Nitrogen Outlet Concentration

**DOI:** 10.3390/plants11111456

**Published:** 2022-05-30

**Authors:** Kwanele Sigcau, Ignatius Leopoldus van Rooyen, Zian Hoek, Hendrik Gideon Brink, Willie Nicol

**Affiliations:** Department of Chemical Engineering, University of Pretoria, Lynnwood Road, Hatfield, Pretoria 0002, South Africa; u16226268@tuks.co.za (K.S.); ignatiuslvr@gmail.com (I.L.v.R.); zianhoek1@gmail.com (Z.H.); deon.brink@up.ac.za (H.G.B.)

**Keywords:** phytoremediation, nutrient pollution, pH control, nitrate removal, *Lemna minor*, nitrogen to proton ratio

## Abstract

Phytoremediation technologies are employed worldwide to remove nutrient pollutants from agricultural and industrial wastewater. Unlike in algae-based nutrient removal, control methodologies for plant-based remediation have not been standardized. Control systems that guarantee consistently low outlet concentrations of nitrogen and phosphorous often use expensive analytical instruments and are therefore rarely viable. In this study, pH measurement was used as the sole input to control the nitrate outlet concentration in a continuously operated *Lemna minor* (lesser duckweed) phytoremediation tank. When grown in 20 L batches of modified Hoagland’s solution, it was found that a constant ratio exists between the amount of nitrate removed and the amount of acid dosed (required for pH control), which was equal to 1.25 mol N·(mol H^+^)${}^{-1}$. The nitrate uptake rates were determined by standard spetrophotometric method. At critically low nitrate concentrations, this ratio reduced slightly to 1.08 mol N·(mol H^+^)${}^{-1}$. Assuming a constant nitrogen content, the biomass growth rate could be predicted based on the acid dosing rate. A proportional-integral controller was used to maintain pH on 6.5 in a semi-continuously operated tank covered by *L. minor*. A nitrogen control strategy was developed which exploited this relationship between nitrate uptake and dosing and successfully removed upwards of 80% of the fed nitrogen from synthetic wastewater while a constant biomass layer was maintained. This study presents a clear illustration of how advanced chemical engineering control principles can be applied in phytoremediation processes.

## 1. Introduction

Nitrogen and phosphorus pollution originating from agricultural and industrial wastewater continue to incur environmental consequences ranging from eutrophication and air pollution to biodiversity loss and climate change [1,2]. Waste discharge is restricted by strict regulations limiting nitrogen and phosphorus concentrations. Therefore, technologies such as reverse osmosis and chemical precipitation methods are employed to remove nitrogen and phosphorus from these wastewaters [3]. Biological methods such as constructed wetlands are considered to be ecologically friendlier and have gained increasing attention [4,5]. The use of aquatic macrophytes—such as *Lemna minor* L. (lesser duckweed)—has proved to be effective at reducing nitrogen and phosphorus concentrations to within environmentally safe limits [6,7]. Fast pollutant removal rates and good process efficiency has been achieved in these systems [8,9,10].

However, current trends in phytoremediation technologies indicate room for improvement. Constructed wetlands (CWs) and macrophyte-based wastewater stabilisation ponds (MWSP) are the two most common configurations employed. In terms of operation, both configurations achieved efficient nutrient polishing. CWs are commonly designed to facilitate phytoextraction of throughput at a certain flow rate by a polyculture of plants. Studies researching MWSPs tend to assess the use of plant monocultures for water treatment. Biological oxygen demand (BOD) reduction tends to be higher in these systems than in CWs. Of the plants frequently studied, duckweed and *Eichhornia crassipes* (water hyacinth) commonly achieve BOD reductions of 52–70% [11,12,13,14]. Water hyacinth has been reported with removal rates of 60–83% nitrate for groundwater with the loading of up to 300 mg·L${}^{-1}$ [11,12]. Fang et al. [13] showed that water hyacinth reduced ammonium-nitrogen and total nitrogen concentrations from 0.25 to 0.05 mg·L${}^{-1}$ (74 days) and from 1.7 to 0.5 mg·L${}^{-1}$ (44 days) respectively in nitrogen-polluted eutrophic pond water.

The biomass of duckweed compared to water hyacinth can be reclaimed more cheaply and more easily due to its lower fibre and lignocellulose content [15]. Körner and Vermaat [14] reported removal rates by duckweed of between 50% and 95% and upwards of 82% and 100% for ammonium and nitrate removal, respectively. Removals of over 70% and 90% in Total Kjeldahl nitrogen and ammonia, respectively [16], and 68% of nitrate removal was achieved with *L. minor* while in combination of *Pistia stratiotes* (water lettuce) [17]. A monoculture of *L. minor* achieved similar nitrogen removal rates of 63.2% while phosphate removal was reported to be significantly lower at 36.2% total phosphate [18]. Abiotic variables also have an influence on the growth of duckweed which in turn affects the rate of nitrogen assimilation. Temperature and light intensity have been reported to have a proportional relationship to the growth rate of duckweed by Lasfar et al. [19]. An oversupply of nutrients have also been shown to inhibit growth. High nitrogen and phosphorus concentrations above 40 mg·L${}^{-1}$ and 15 mg·L${}^{-1}$ respectively have been shown to inhibit growth rate [19]. Upon exposure to toxic ions such as ${\mathrm{Ag}}^{+}$, *Lemna gibba* have measured to have inhibited growth due to hyper-accumulation of the ion [20,21,22].

The previously mentioned literature shows that phytoremediation has the potential to innovate water treatment technologies, however scaling of these technologies remain elusive; all of these results were obtained in batch systems. Of the literature surveyed, none of them detailed how traditional phytoremediative techniques have been adapted to continuous operation. There appears to be a knowledge gap when it comes to continuous bioreactors using plants and how online measurement of process variables improve water quality. Improved control systems have been directly related to increased productivity and higher overall nitrogen removal. However measurement instruments like ion-selective-electrodes and sophisticated analysis methods such as elemental analysers although beneficial are expensive [23,24,25].

Even though online control of the phytoremediative water treatment systems remains highly conceptual, various studies have established the precedent for online measurement to control conditions such that optimal nutrient removal can be achieved. Within these, algal treatment methods are common. Emphasis is often placed on maximising biomass production which is directly linked to nutrient removal [26]. Mcginn et al. [26] was able to control the outlet flowrate of a micro-algae photo-bioreactor (and thus the dilution rate) using the biomass concentration as input. The biomass concentration was measured by dual excitation fluorometer developed in-house which was based on spectrophometer readings. In the method described, Franca et al. [27] had great success in inferring the CO${}_{2}$, NO${}_{3}$, and total P concentrations. By using spectrophotometric measurements as input for the inferences, it was possible to manipulate the feed rate of CO${}_{2}$ and control nitrogen and phosphorus concentrations. A major disadvantage of these systems is that they are suited to a very specific reactor configuration which makes use of inline spectrophotometry. As such, these methods cannot be adapted for use in open ponds similar to wetlands and are incompatible with plants typically used in wetlands.

The hypothesis proposed in the current study is that pH can be used as the sole input variable for controlling the nitrate concentration in the discharge stream of a phytoremediation system. pH is a reliable and relatively cheap measurement and would provide a viable control option. This hypothesis relies on the fact that when nitrate is absorbed and assimilated by plants, alkalisation of the aqueous medium occurs from the release of OH${}^{-}$ ions, and the co-absorption of H${}^{+}$ ions [28,29,30,31,32]. Consequently, pH changes in the medium are related to the amount of nitrate absorbed by the plants.

In this study, this would directly relate to the control of the nitrate concentration in the effluent of a *L. minor* water remediation tank. To test this a nitrogen remediation study was performed in a semi-continuously operated system, where the pH characteristics of the system were used to manipulate the input of synthetic wastewater. The relationship between growth, nitrate uptake and pH dynamics were established. The pH-nitrate-growth characteristics were incorporated in the feed algorithm of the semi-continuous system to control nitrate breakthrough and biomass removal. Ultimately, the wastewater throughput was manipulated as a function of the varying nitrogen removal characteristics of the pond.

## 2. Materials and Methods

### 2.1. Methods and Planning

Non-axenic *L. minor* culture was obtained from the botanical gardens and greenhouses at the University of Pretoria (S 25°45′21″ E 28°13′51″). Plants were cultured in-house on modified hydroponic growth solution in 20 L and 40 L basins under lighting with photo-period of 16 h. A rectangular tank (80 cm × 51 cm × 9.8 cm) was filled with hydroponic medium. On the liquid surface, *L. minor* plants were grown. Plants were grown exposed to open air and grown under light. The water level reduced by evaporation was restored by addition of de-ionised water. For all experimental runs, pH liquid environment was measured continuously and controlled. Abiotic conditions of the experiments such as light intensity and temperature were constant. Water temperature was measured throughout the duration of the batch (thermometer) and continuous runs (water sensor) and found to be the same as the air temperature. Regular measurements showed a slight deviation from the mean temperature of 22 °C by maximum of only 2 °C.

A photoperiod of 16 h was used for the batch runs as well as the stock culture of *L. minor*. A modified hydroponic medium circa 10% dosage of full Hoagland’s medium [33] was prepared, composed of 50.5 mg·L^−1^ KNO_3_, 118 mg·L^−1^ Ca(NO_3_)${}_{2}$· 4H_2_O, 123.25 mg·L${}^{-1}$ MgSO${}_{4}$· 7H_2_O, 13.6 mg·L${}^{-1}$ KH${}_{2}$PO${}_{4}$, 2.25 mg·L${}^{-1}$ Fe-NaEDTA, 0.286 mg·L${}^{-1}$ H${}_{3}$BO${}_{3}$, 0.008 mg·L${}^{-1}$ CuSO${}_{4}$· 5H${}_{2}$O, 0.181 mg·L${}^{-1}$ MnCl${}_{2}$· 4H${}_{2}$O, 0.022 mg·L${}^{-1}$ ZnSO${}_{4}$· 7H${}_{2}$O, and 0.012 mg·L${}^{-1}$ NaMoO${}_{4}$· 2H${}_{2}$O. Run 1 and Run 2 were inoculated with duckweed such that the tank was only partially covered with biomass. At partial coverage, analysis of images taken was used to quantify the biomass in the tank. The amount of green colour that appeared in aerial image of the tank surface analysed using a K-means cluster counting method [34]. The imaging measurement was used as a comparison to the acid dosing based measurement. Run 1 was inoculated with 11.65 g, with a surface area coverage of 23.4%. Run 2 was inoculated with 26.9 g, with surface area coverage of 61.2%. Growth of *L. minor* at partial surface area coverage was compared to the growth at visibly full surface area coverage. In Run 3, 57.24 g of biomass inoculated the tank, where the tank’s biomass density was measured via regular physical representative measurements.

Subsequent experiments testing the removal efficiency of a nitrogen removal strategy which was meant to control the nitrogen concentration in the tank effluent. The pH controller was used to infer when nitrogen levels were low. For consistent and easier control, the photo-period was extended to 24 h and a 9400 lux lamp was used supplied light. The hydroponic medium used was 10% dosage of full Hoagland’s medium except for nitrogen which was lower than normal tenth dosage; composed of 123.25 mg·L${}^{-1}$ MgSO${}_{4}$ · 7H${}_{2}$O, 13.6 mg·L${}^{-}$ KH${}_{2}$PO${}_{4}$, 2.25 mg·L${}^{-1}$ Fe Na-EDTA, 0.286 mg·L${}^{-1}$ H${}_{3}$BO${}_{3}$, 0.008 mg·L${}^{-1}$ CuSO${}_{4}$ · 5H${}_{2}$O, 0.181 mg·L${}^{-1}$ MnCl${}_{2}$· 4H${}_{2}$O, 0.022 mg·L${}^{-1}$ ZnSO${}_{4}$· 7H${}_{2}$O, 0.012 mg·L${}^{-1}$ NaMoO${}_{4}$· 2H${}_{2}$O and 13.48 mg·L${}^{-1}$ KNO${}_{3}$, 31.487 mg·L${}^{-1}$ Ca(NO${}_{3}$)${}_{2}$· 4H${}_{2}$O as well as 197.09 mg·L${}^{-1}$ KCl and 388.81 mg·L${}^{-1}$ CaCl${}_{2}$· 2H${}_{2}$O. Macro- and micro-nutrients were replenished by small doses of 300% strength Hoagland’s medium. In Run 6, the aforementioned medium was used with an additional 71.82 mg·L${}^{-1}$ hydrogen peroxide (2.11 mM) for algal control in the medium supply.

### 2.2. Apparatus and Analytical Methods

Level control and pH control were facilitated by an Arduino MEGA 2560${}^{™}$. Measurements were taken using an Analog Haoshi H-101 pH meter pro and logged regularly. The pH was adjusted with the addition of 0.1 M hydrochloric acid solution using a stepper motor peristaltic pump. Water and tank purge was done using a Flojet LFP model 12 V diaphragm pump Xylem${}^{™}$ (Rye Brook, New York, USA). For the liquid medium, chemicals were sourced from Merck${}^{™}$ (Darmstadt, Germany) (purity of 98%).

Analysis of the nitrates was done on liquid samples using Spectroquant 0.10–25.0 mg/L NO${}_{3}$-N Nitrate Cell Test and Spectroquant 23–225 mg/L NO${}_{3}$-N Nitrate Cell Test photometric methods from Merck${}^{™}$. Reported nitrate values were averages of three repeat tests on the same sample. Sampled values were calibrated for 340 nm wavelength spectrophotometer measurements (Agilent Technologies, Santa Clara, California, USA, Cary 60 UV-Vis, G66860A).

**Figure 1:**
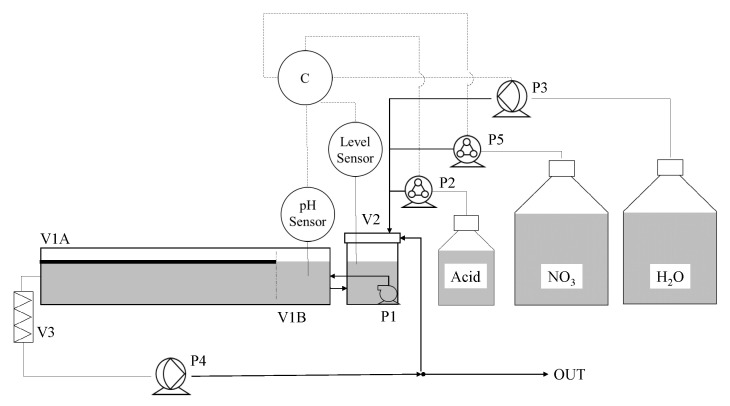
Diagram of phytoremediation tank. P1 is a submersible pump, P2 and P5 are stepper motor peristaltic pumps, P3 and P4 are larger diaphragm pumps. V1 is a 30 L tank: *L. minor* was grown in section A under light and B was kept covered and separate for pH measurement. V2 is a vessel were fluids were introduced and mixed.V3 is a filter used in the outlet tube of the tank. The nitrate (NO3) supply was contained 47 mM nitrate-nitrogen.

Before inoculation of the tank, plants were rinsed, dried for 45–60 min on a paper towel in the open air and weighed. Lighting applied by MarsHydro hydroponic lights. Plants were grown in an area of 0.27 m${}^{2}$. A modified flat fishing net with known dimensions of 70 mm by 92 mm was used for representative fresh weight measurements. At the end of the run, plants were weighed to obtain fresh weight after carefully being removed from the tank and dried for 45 min. After drying for 48 h, the dry weight was obtained. The relative growth rate (*RGR*) was determined using Equation (1) below:

(1) $$RGR=\frac{\ln\left(\frac{M_{f}}{M_{0}}\right)}{t_{f}-t_{0}},$$

where *M${}_{0}$* and *M${}_{f}$* are the measured fresh weight (wet mass) at inoculation and after final removal in grams, and *t${}_{0}$* and *t${}_{f}$* are the times of inoculation and final removal in days. The nitrogen removal in the effluent was reported for the remediation experiment in Run 6. Equation (2) was used to determine the removal of nitrogen. Inlet and outlet flow rates were the same.

(2) $$Fraction\;Removed=\frac{F_{N_{fed}}-F_{N_{measured}}}{F_{Nfed}},$$

where *F${}_{N_{fed}}$* is the molar flow rate of nitrate-nitrogen fed into the tank in mmol·d${}^{-1}$ and *F${}_{N_{measured}}$* is the molar flow rate of nitrate-nitrogen measured in the liquid medium in mmol·d${}^{-1}$. A schematic diagram is given in Figure 1. Due to Run 1, Run 2 and Run 3 being operated in batch, pump P4, filter V3 and the outlet were not used. All instruments were used while operating continuously.

## 3. Results

Plants interact chemically with their environment; pH measurement is just one way to observe some of these interactions. When nitrate is absorbed and assimilated, OH${}^{-}$ exudation occurs which increases the pH of the medium surrounding the roots [29,30,31,32]. Assuming a fixed amount of OH${}^{-}$ exudation occurs per nitrate assimilated, controlling the pH with acid dosing allows for calculation of the amount of nitrate absorbed.

A remediation tank system containing *L. minor* was operated in batch and semi-continuously while the pH was maintained at 6.5 through proportional-integral feedback control. Batch runs were performed to establish a relationship between acid dosed and nitrate absorbed. Specifically, the ratio between the nitrate taken up by *L. minor* and acid dosing required to return pH to the setpoint was studied. In the semi-continuous runs the study aimed to reduce the nitrate concentration in the effluent. The relationship between the nitrate and acid dosing was especially important in expressing the nitrate removed in terms of biomass quantification.

Run 1 and Run 2 were inoculated with duckweed such that the tank was only partially covered with biomass. As shown in Table 1, Run 1 was inoculated with 11.65 g, with a surface area coverage of 23.4% and was terminated when the tank had been fully covered. Run 2 was inoculated with 26.9 g, with surface area coverage of 61.2%. Run 2 was not terminated at full coverage and allowed to grow until nitrates were exhausted. At partial coverage image analysis algorithm was used to quantify the spread of fronds in the tank and serve as a comparative quantification of the biomass. From overhead images taken of the liquid surface, the analysis algorithm detected the amount of green pixels relative to all the pixels in the image.

The tank was found to be fully covered when biomass was greater than 200 g·m${}^{-2}$ or 49 g. In Run 3, 57.24 g of biomass was added initially and the biomass density in the tank was measured throughout the run. It was thought that physical measurements could be used as a representative estimate of the density over the entire tank. In order to approximate the biomass mat density in the tank, biomass was physically removed from a sample area of 63.0 cm${}^{2}$, weighed and replaced. The pH was controlled at the same setpoint of 6.5 for all runs. The initial and final biomass measurements as well as the relative growth rates (*RGR*) are given in Table 1.

### 3.1. Determination of Nitrate-Proton Ratio and Biomass Quantification in Batch-Operated System

**Figure 2:**
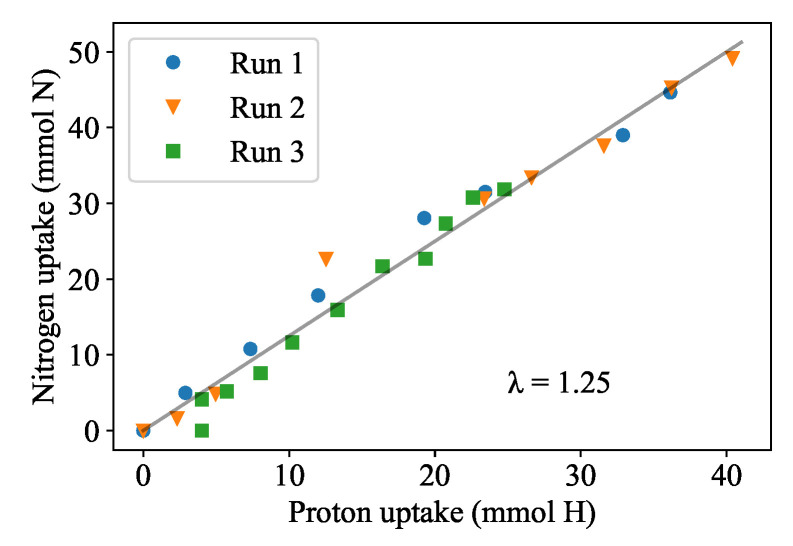
Results from batch experiments in 10% Hoagland’s medium where the pH was controlled at 6.5. The relationship between absorbed nitrogen and dosed protons (*λ*) was determined to be the same value of 1.25 mol N·mol^−1^ H as indicated by the common slope.

Figure 2 compares the nitrate uptake rate to the proton (acid) dosing rate for Runs 1, 2 and 3. A constant ratio of the nitrate uptake and acid dosing (λ) of 1.25 mol N·(mol$\;H^{+}$)${}^{-1}$ is observed as the slope of Figure 2. With this relationship, the proton dosing rate ($D_{R}$) provides an estimate of the nitrate demand and an indication of the duckweed’s instantaneous growth rate (assuming constant biomass nitrogen content). The biomass production rate can then be calculated by multiplying the proton dosing rate with λ and dividing by the nitrogen content in the biomass, which was measured at 61.9 mg N·g${}^{-1}$ dry mass. The water content of the duckweed was measured at 0.902 g·g${}^{-1}$.

**Figure 3:**
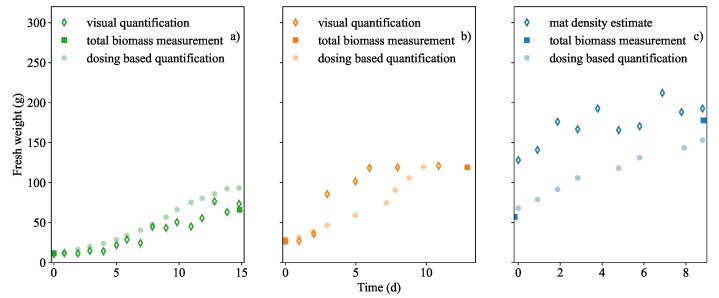
Biomass quantification methods were compared. Visual based estimation (photo pixel analysis) of the biomass coverage in (**a**) Run 1 and (**b**) Run 2. Biomass density determinations were based on physical mass measurements in a known area in (**c**) Run 3. Additionally, biomass quantification based on the acid dosing was included in (**a**–**c**). All measurement techniques were compared against the available initial and final actual mass measurements.

At partial coverage, a comparison between the visual-based quantification and acid dosing based quantification was made which is shown in Figure 3a,b. At full coverage the visual quantification was unable to accurately measure submerged biomass, thus the acid dosing based quantification was compared to the measured mat density which is shown in Figure 3c.

### 3.2. Automated System for Nitrogen Effluent Minimisation in Semi-Continuous Operation

To operate the photremediation tank continuously, a control strategy was developed to exploit the nitrogen-related pH behaviour. This algorithm was based on the work of van Rooyen and Nicol [35] and was designed to clean nutrient-polluted water, simulated by 10% strength Hoagland’s growth solution [33] and remove the nitrogen to achieve a nitrate concentration lower than 0.05 mM in the effluent water. The focus of this study was nitrate-nitrogen removal, therefore, other macro-nutrients were required in excess such that nitrogen would be limiting. Biomass was harvested regularly to keep the biomass density fairly constant.

**Figure 4:**
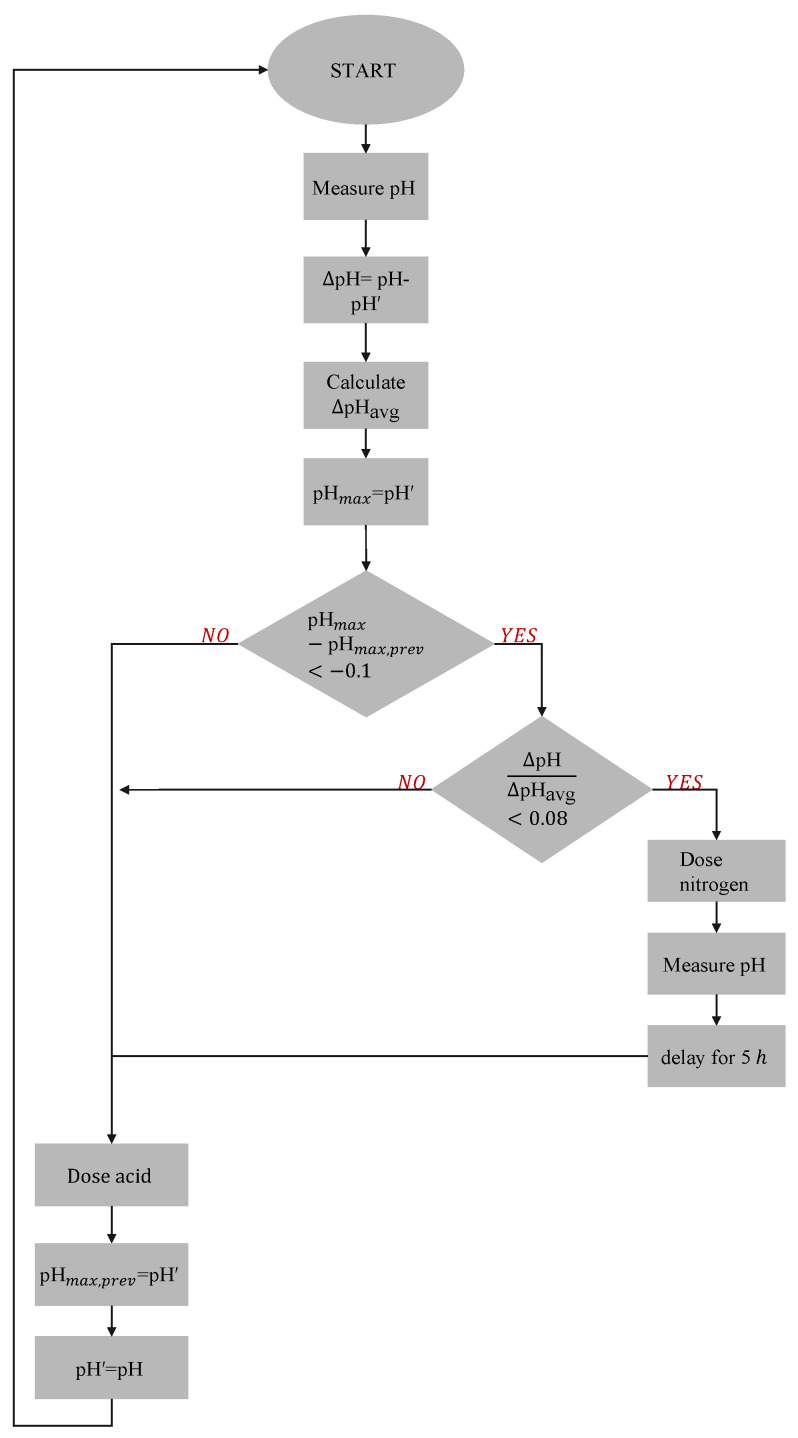
Control algorithm for detection of low nitrogen concentration. The pH was controlled by acid dosing according to a standard proportional-integral control algorithm. pH measurements were taken every 30 min (sampling time) and acid dosing occurred every 60 min (amounts dictated by the control algorithm). The slope between the pH measurement 30 min after an acid dosing instance and the pH immediately before to next acid dosing instance was recorded. A reduction in this slope indicates a reduction in the hydroxide exudation (nitrate uptake) of the duckweed. The ratio between this slope (Δ*pH*) and the running average of these slopes (ten previous values) (Δ*pH_avg_*) was used as an indication of nitrate extinction. If this ratio (Δ*pH*/Δ*pH_avg_*) fell below a threshold of 0.08 (*ϵ*), the nitrogen was too low and assumed to be exhausted. Thereafter a pump was turned on to feed wastewater (containing nitrate) into the tank only after a reduction in the pH maxima (*pH_max_*) slopes greater than 10% over time.

The control scheme for the detection of a low nitrogen concentration in the tank outlet is shown in Figure 4. The pH control relied on measuring pH between a rising slope and a descending slope. pH samples were taken 30 min apart. When a descending slope occurred between two pH readings, it was caused by an acid addition proportional to the difference between the measured pH and the setpoint. Between two pHs on the rising slope, no acid additions are made and the change in the pH was a result of the uptake by duckweed. For the detection of nitrogen depletion, the ratio of the absolute pH difference on the rising slope and the average pH change (Δ*pH*/Δ*pH_avg_*) was calculated and was used as a criteria for detecting when to replenish the nitrogen supply. The average pH change was based on a running average of ten pH maxima values and was updated every hour, except when dosing nitrogen. At N-depletion, a pump was turned on to feed into the tank. To avoid false indications, the control would only act if there was more than 10% reduction in the previously measured pH maxima (between a decreasing slope and rising slope).

**Figure 5:**
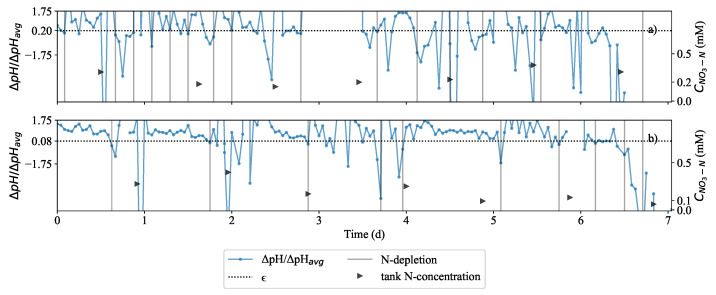
Comparative plot of experiment where the ratio between the absolute change in pH and the running average of absolute pH change (Δ*pH*/Δ*pH_avg_*). The threshold for this ratio was set to a value referred to as the depletion threshold (*ϵ*): 20% (**a**) and 8% (**b**). In (**a**) and (**b**) Δ*pH*/Δ*pH_avg_* was plotted for each case. When Δ*pH*/Δ*pH_avg_* dropped below *ϵ*, this was the point where the system indicated nitrogen depletion below detectable concentration for the plant. The nitrate-nitrogen concentrations were observed to be decreasing over time until minimum nitrate level was reached, 0.22 mM in (**a**) and 0.061 mM in (**b**) medium nitrate concentration despite regular feeding of fresh medium dosed at the times indicated on the plot.

Figure 5 shows exploratory implementation of control where the nitrogen removal was compared at two different depletion thresholds (ϵ): 0.08 and 0.20. The same starting biomass amount of 282 g was used in Run 4 and Run 5 while all other conditions were the same. There was a nitrogen depletion (indicated by gray vertical lines) when Δ*pH*/Δ*pH_avg_* value decreased below 0.20 in Figure 5a in Run 4 and 0.08 in Figure 5b in Run 5. This showed that there was a significant difference in the effluent nitrogen concentration at ϵ of 0.2 compared to ϵ of 0.08. Due to the higher ϵ in Figure 5a than in Figure 5b, the time between depletion instances was shorter.

### 3.3. Implementing the Control System for Continuous Nitrate Removal

**Figure 6:**
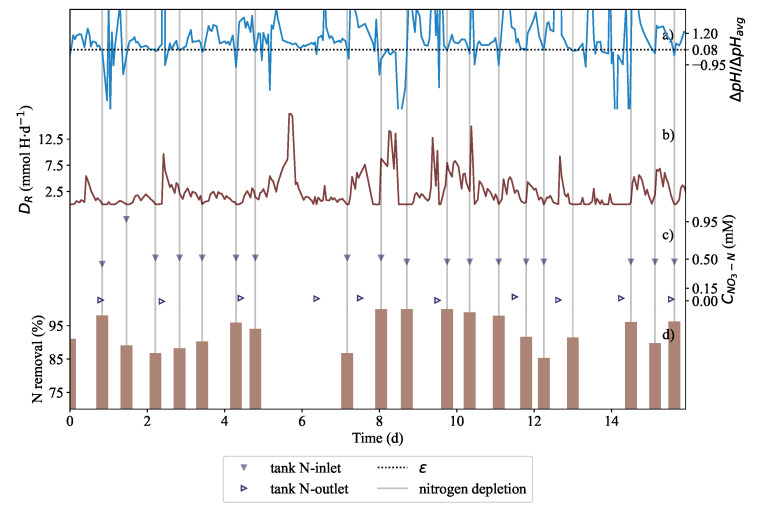
Results showing the nitrate-nitrogen removal in the phytoremediation tank. In subplot (**a**), showing Δ*pH*/Δ*pH_avg_*, at a 92% reduction or at the chosen *ϵ* of 0.08, nitrate was assumed to be depleted (vertical lines are depletion instances and also show when nitrogen was fed). (**b**) The rapid drops in *DR* correspond to the depletion of nitrate. As soon as nitrate was fed into the tank, *DR* rapidly increased. (**c**) Nitrate measurements showed that effluent nitrogen was maintained at critically low concentrations. Synthetic wastewater was fed into the tank containing nitrate at depletion/dosing instances. The inlet concentration of the feed is included along with the effluent concentration. (**d**) Shown is the total percentage of nitrogen removal by *L. minor* measured before nitrogen was dosed. The phytoremediation system achieved a high fraction of removal almost regularly every 10–14 h.

In Run 6, continuous operation was attempted based on the exploratory run reported in Figure 5. The inoculation mass was 286 g of *L. minor* obtained from a stock culture grown in full 10% Hoagland’s solution at 16 h photoperiod. After inoculation, the duckweed was left in the hydroponic medium for the first 110 h of the run to help acclimatize the plants after transfer until the first nitrogen depletion at time zero in Figure 6. Whenever all available nitrogen was exhausted, the feedback proportional-integral controller instructed the pump, P5 (Figure 1), to supply fresh 10% Hoagland’s medium to restore the concentrations of all of the nutrients.

In Figure 6a, Δ*pH*/Δ*pH_avg_*, is shown and DR was reported in Figure 6b. The rapid drops in Δ*pH*/Δ*pH_avg_* corresponded to the vertical lines which indicated when depletion occurred in Figure 6 detected after the pH slopes had decreased 92% relative to the running average. It is shown in Figure 6c that nitrate concentration was measured to be very low while in Figure 6d, the corresponding nitrate removal was calculated. The values presented in the figure should be interpreted as: of the nitrates that are fed into the reactor the nitrate removal represents the fraction of the nitrates removed from the throughput. An average throughput rate of 7.2 L·d${}^{-1}$ was passed through the remediation system with a retention time of 2.96 days.

**Figure 7:**
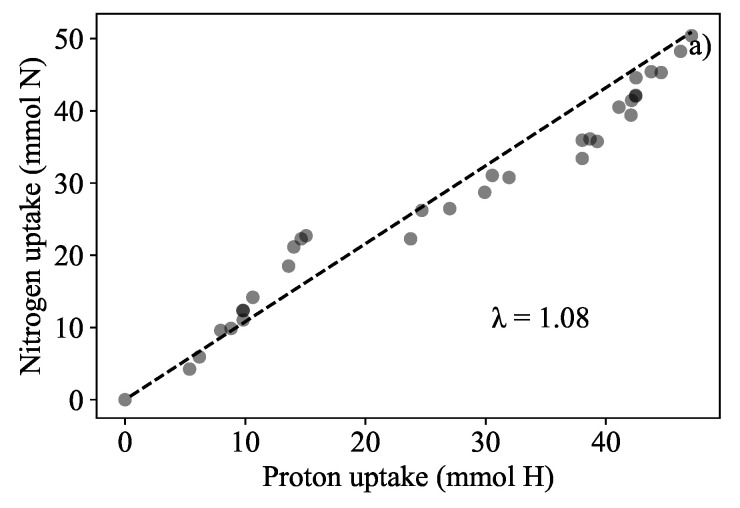

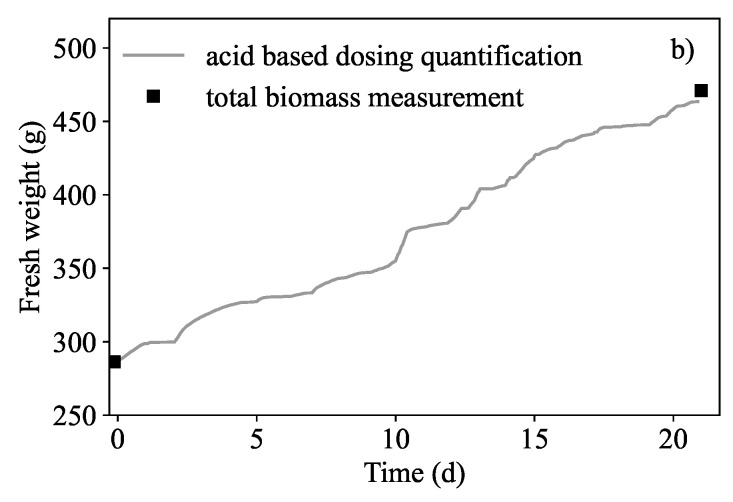
Growth quantification results in the semi-continuous run. (**a**) The relationship between absorbed nitrogen and dosed protons (*λ*) was re-determined to be 1.08 mol N·(mol$\;H^{+}$)${}^{-1}$ indicated by the slope. Initial and final biomass measurement were 286 g and 470 g respectively. (**b**) Biomass prediction of biomass to show mass gained from the uptake of nitrogen with a percentage error of 2.37%.

The total biomass amounted to 474.66 g wet mass which was an increase of 65.96% (compared to the starting biomass of 286 g which amounted to a relative growth rate of 0.017 d${}^{-1}$). It was found that the total nitrate nitrogen removed from the liquid amounted to 59.39 mg NO${}_{3}$-N·g${}^{-1}$ dry biomass and the nitrogen removed by the biomass was estimated to be 61.90 mg NO${}_{3}$-N·g${}^{-1}$ dry biomass. In addition, it was possible to show that biomass in the continuous system could also be predicted. Acid dosing based biomass quantification could be used to infer the amount of nitrogen extracted from the medium. This was determined similarly to how the acid dosing based prediction was found for the batch runs reported in Figure 3. A λ-value of 1.08 mol N·(mol$\;H^{+}$)${}^{-1}$ was found to fit better for this biomass prediction in Figure 7a. The prediction of the new biomass growth is presented in Figure 7b. The error between the dosing estimation and the measured biomass was 2.37%.

## 4. Discussion

### 4.1. Biomass Quantification Using Acid Dosing Compared to a Visual-Based Quantification and a Representative Mat Density Quantification

*L. minor* is widely known to have a vegetative growth pattern in a fashion similar to many bacteria and divide from a mother frond into at least two daughter fronds (a frond is an individual unit composing of a leaf and smaller roots) [36,37,38,39]. It is understood that duckweed biomass increases in two ways. When partially covered, large open spaces exist between clusters of fronds. Growth in this regime was associated with an increase in the surface area coverage. The mat of *L. minor* would increase in thickness only after the liquid surface had been completely covered. This regime was referred to as fully covered. It was assumed that no increase in the biomass mat thickness occurred at partial coverage and once fully covered, there was no change in the amount of surface area coverage.

In Run 1 the visual biomass quantification method served as a good comparison to acid dosing quantification. It appeared that acid dosing based quantification was the least accurate of the two biomass prediction methods in Figure 3a. The trend was similar to that of the acid dosing based quantification. This was especially true when the visual prediction occurred within the calibration limits and the output visual analysis algorithm did not seem to be limited by the movement or displacement of biomass fronds.

However in Run 2 (Figure 3b), it is shown that the visual method was insufficient in detecting growth beyond the full capacity of the tank surface. Hence by using the visual imaging method, growth at partial coverage appears to halt suddenly at 50–53 g. When compared to the acid dosing based quantification in the same figure, it is shown to be the least accurate of the two prediction techniques. The dosing-based quantification more closely followed the trend of growth until dosing stopped at nitrate extinction. Dosing also confirmed that maximum growth occurred after 6 days as indicated by the curve inflection in Figure 3b. In the figure, it was necessary to re-adjust the calibration and extrapolate the visual estimate because growth had exceeded full capacity. Any additional discrepancy between the visual quantification and dosing estimate can be attributed interference from an additional shade of green from an algal infection. Growth above 200 g·m${}^{-1}$, (49 g) caused fronds to overlap and as a result, increases in the duckweed mat density was undetectable. This included any additional biomass that was not visible like the roots which appeared to grow longer.

It was observed from Figure 3c that mat density was not the same over the entire surface of the tank and as a result the estimation over-predicted the biomass initially. It was more accurate when the biomass density in the tank became higher. Thus, it was concluded that the method was only useful when the tank was very dense. As a comparative quantification method, one could not rightly say that a representative mat density measurement is more useful than simply weighing all the biomass repeatedly, however this too presents its own problems such as non-negligible mass losses. Thus Figure 3c shows that acid dosing based estimate is a good prediction at higher mat densities.

The dosing based estimation was concluded to be a realistic representation of the growth trend. This was because it was based on the nitrate uptake by *L. minor* and could be accurately used regardless of full or partial coverage and it was not necessary to extrapolate for extremely dense biomass mats.

### 4.2. Selection of ϵ to Operate at Critically Low Nitrate Concentrations

In Figure 5b, ϵ of 0.08 was found to work better than ϵ of 0.2 because the effluent could be maintained at a very low nitrogen concentration. This was the case in Run 5 (Figure 5b) where the effluent nitrogen concentration could be controlled between 0.05 mM and 0.15 mM. This was significantly lower than in Run 4 (Figure 5a), where the effluent nitrogen concentration could be contolled between 0.15 mM and 0.30 mM. As can be seen in the first two days, Δ*pH*/Δ*pH_avg_* value is noisy. The condition of a 10% reduction in the pH maxima was therefore necessary. This would prevent erroneous dosing instances after the second day. Van Rooyen and Nicol [35] explained that Δ*pH*/Δ*pH_avg_* approached zero as more nitrates were consumed by *Brassica oleracea* which showed that effective control could be achieved at desired nitrate concentrations to maintain a healthy growing environment for the plant. It was preferred that ϵ remained 0.08. The authors hypothesised that the same tight control could be applied to keep the nitrate concentration very low. Shown in Figure 6c, the remediation system was operated at a extremely low nitrogen concentrations; concentration at various depletion instances was practically zero. This is further supported by sharp decline of pH which resulted in a reduction of *D_R_*. It is believed that this corresponded to a decrease in exudation discussed by Dijkshoorn [31] and Tischner [30] as responsible fo alkalisation of the liquid. It was understood that the lower rate of alkalisation in the medium implied that the growth was slower. Δ*pH*/Δ*pH_avg_* in Figure 6 demonstrates that pH is a very dynamic response. This means that there was an insignificant amount of time between feeding more solution and for Δ*pH* to increase. As long as *L. minor* was receiving sufficient nitrogen, the pH would always increase. Over time, the running average Δ*pH*/Δ*pH_avg_* would also grow larger. Eventually Δ*pH*/Δ*pH_avg_* would become very large.

### 4.3. The Trade off between High Nitrate Removal and Growing Speed in an Automated Nitrogen Removal System

Nitrate depletion was detected approximately every 10–14 h. In Figure 6c, nitrogen in the tank varied between 0.0 mM and 0.30 mM with an inlet nitrogen feed concentration ranging between 0.5 mM and 1 mM. Due to the system operating until depletion, the treated water effluent could be discharged at low outlet concentration. Therefore the removal of nitrate was dependent on *L. minor*’s uptake characteristics. The 80% nitrate removal efficiency was an indicator of good performance in the system despite a slow growth rate. This result is comparable to that of Körner and Vermaat [14], Alaerts et al. [16], and Ayyasamy et al. [11]. Nitrate measurements and nitrogen removal data in Figure 6d confirmed that disruptions at 5–7 days did not effect the efficiency of removal severely.

There seemed to be a trade-off between the high nitrate removal and the growth rate. Under nutrient sufficient conditions, *L. minor* is able to grow relatively fast as indicated by Bian et al. [20]. There appears to be a minimum nitrogen concentration such that the growth rate is optimum. The medium contained an initial concentration of 0.40 mM NO${}_{3}^{-}$ while traditional nutrient media have a nitrogen concentrations of between 5 mM to 15 mM [20,33]. Although the system was able to operate under nitrogen-lean conditions, it was observed that there was slow biomass growth which was likely a stress response to the nitrogen. As such, it could be said that high nitrogen removal efficiency was prioritised at the cost of fast biomass growth. As previously discussed, one could infer this observation from the dosing rate in Figure 6b. There was a sharp decrease in $D_{R}$ as the system approached nitrate depletion. This resulted in short periods of zero to very little dosing. As soon as the medium was fed, dosing increased again. Appenroth et al. [40] suggests that a physiological dormant response of *Spirodela polyrhiza* was positively associated with low nitrate concentrations and that turion germination could be stimulated by the presence of nitrate. As such, low $D_{R}$ was probably an indicator of sluggish activity, however this was not examined in depth.

An observed increase of 188.66 g in the biomass was measured which corresponded to an increase of 65.96% of the inoculation mass over the course of 21 days. Under normal conditions, this would be considered very slow production. The relative growth rate of 0.017 d${}^{-1}$ was ten times lower than in previous batches (Table 1). Higher growth rates have been associated with nutrient removal [1,6,7,18,25,26]. Ultimately, the lower yield could be considered a consequence of the nitrogen-limitation stress. At such a low nitrogen supply, this was to be expected. The stock culture of *L. minor* was prepared with nitrogen supply of 1.5 mM NO${}_{3}^{-}$ while nitrogen in the tank varied between 0.01 mM to 0.3 mM.

**Figure 8:**
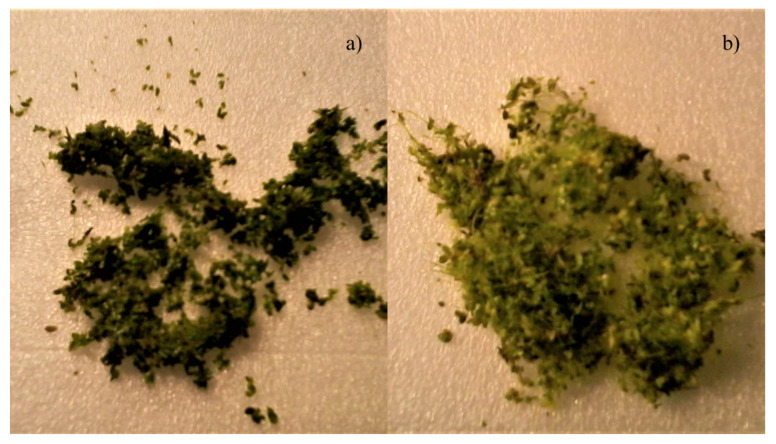
Images of *L. minor* from (**a**) before the run, fronds of the plant have very short or non-visible roots and are dark green in colour compared to the plant, and (**b**) after the run where fronds are lighter green in colour and roots are longer (vary between 1.5 cm and 4 cm). Individual fronds clumped together by longer roots.

It was also observed that the rate of nitrate uptake increased as compared to just after inoculation. Within the first 100 h after inoculation, the absorption rate of NO${}_{3}^{-}$-N was found to be 0.0144 mmol·g${}^{-1}$·d${}^{-1}$. After 16.5 d, the rate had increased to 0.0927 mmol·g${}^{-1}$·d${}^{-1}$ which demonstrates a higher demand for nitrogen, possibly due to nitrogen-lean stress mechanisms of duckweed. Root growth was also observed in Figure 8. It was thought that the development of dense roots from fronds occurred as physiological response to nitrogen limitation thus affecting the uptake rate. Cedergreen and Madsen [28] noted their observation of root growth of duckweed having a linear proportionality to the ammonium and nitrate uptake rate. Although the literature referenced in the study [28,36,37,40] mention that the carbon to nitrogen ratio within the biomass tended to increase, the present study cannot say whether there was a change to the elemental composition within the biomass, further work is required to confirm this.

### 4.4. Dosing-Based Biomass Quantification Measuring Nitrogen Removal

A prediction error of 2.37% confirmed that the dosing biomass prediction method was sufficiently accurate. Nitrogen removal could be quantified in terms of biomass production (59.39 mg NO_3^−^_N·g${}^{-1}$ dry biomass nitrate removal compared to an estimated change in biomass nitrogen of 61.90 mg NO_3^−^_N·g${}^{-1}$ dry biomass).

It was found that when using the pre-established λ of 1.25 mol N·(mol$\;H^{+}$)${}^{-1}$, biomass was over-predicted the actual measurements and the error of prediction was significantly larger than that of Figure 7a. The nitrogen to proton ratio was recalculated for Run 6 and a λ value of 1.08 mol N·(mol$\;H^{+}$)${}^{-1}$ was found, this value was used instead to predict biomass growth. Λ was observed to decrease in the continuous system (medium dosed all other nutrients were supplied in excess except for the nitrogen). The controller dosed more protons than nitrates that were taken up. The authors surmise that nitrate uptake was affected by nitrate availability. Although it is currently unknown to what extent the other nutrient ions (calcium, magnesium, potassium, phosphate, and sulphate) contributed to λ in both in nitrate-sufficient and nitrate-limited conditions, it is believed that nitrate had the largest affect on λ [30,41]. The authors surmise that the slow growth of *L. minor* was related to the reduction in λ.

## 5. Conclusions

The work presented above has demonstrated the validity of the proposed hypothesis that just by using pH as an input, it was possible to eliminate the nitrogen from wastewater in a continuous phytoremediation system involving *L. minor*. After quantifying the pH-nitrate-growth characteristics, pH could be used to infer the duckweed growth based nitrogen uptake. Biomass growth was predicted based on the acid dosing which most accurately quantified biomass regardless of whether duckweed partially covered the surface or fully covered the surface. Acid dosing based quantification was a non-destructive technique and could be used as an online measure of biomass. Therefore dosing was a better quantification technique than measuring the biomass mat density or a visual-based biomass estimation method. The acid dosing corresponded to the amount of nitrate absorbed by *L. minor*. The study demonstrated a unique method of nutrient removal from water using *L. minor*. Just by using the pH as an input variable, the nitrate nitrogen concentration in effluent was controlled using a proportional-integral feedback control scheme. This was due to the ability of the system to discharge water as soon as it detected when nitrogen had been depleted. This achieved sufficiently high throughput of treated water of 7.2 L·d${}^{-1}$ and high nitrogen removal rates of over 80%. It was found that a high nitrogen removal was obtained at the cost of growth as *RGR* showed a decrease of 90%. The authors recognise that in a larger system, the degree to which mixing occurs may reduce the accuracy of pH and nitrate measurements. Thus a system with sufficient mixing is necessary for consideration. The work was solely conceptual. As such, there is no direct implementation yet.

## Figures and Tables

**Table 1:** Starting biomass and final biomass measurements for batch runs: Run 1, Run 2 and Run 3.

	Initial (g)	Final (g)	*RGR* (d${}^{-1}$)
Run 1	11.65	65.97	0.11
Run 2	26.90	119.5	0.112
Run 3	57.24	177.9	0.125

## Data Availability

The data presented in this study are openly available in the University of Pretoria Research Data Repository at DOI: 10.25403/UPresearchdata.19908076.

## Abbreviations and Symbols

The following abbreviations and symbols are used in this manuscript:

CW	Constructed Wetland
MWSP	Macrophyte-based wastewater stabilisation pond
RGR	relative growth rate (d${}^{-1}$)
λ	nitrogen to proton ratio (mol N·mol${}^{-1}$ $H^{+}$)
Δ*pH*	absolute pH change
Δ*pH_avg_*	running average of pH change based on ten values
Δ*pH*/Δ*pH_avg_*	ratio of absolute pH change and average pH change
ϵ	ratio of pH-uptake reduction (pH units · pH units${}^{-1}$)
